# Retraction Note: TGFβ3 recruits endogenous mesenchymal stem cells to initiate bone regeneration

**DOI:** 10.1186/s13287-022-03108-3

**Published:** 2022-08-05

**Authors:** Moyuan Deng, Tieniu Mei, Tianyong Hou, Keyu Luo, Fei Luo, Aijun Yang, Bo Yu, Hao Pang, Shiwu Dong, Jianzhong Xu

**Affiliations:** 1 National & Regional United Engineering Lab of Tissue Engineering, Department of Orthopaedics, Southwest Hospital, the Third Military Medical University, Chongqing, China; 2 Department of Biomedical Materials Science, College of Biomedical Engineering, Third Military Medical University, Chongqing, China; 3 Department of Surgery, Fuzhou Mawei Naval Hospital, Fujian, China

## Retraction Note to: Stem Cell Research & Therapy (2017) 8:258 https://doi.org/10.1186/s13287-017-0693-0

The Editors-in-Chief have retracted this article. After publication, concerns were raised regarding potential image duplication in the figures.

Specifically:

- In Fig. 1b, the 0 ng + SB and 25 ng + SB group images appear to originate from the same sample (rotated 90 degrees);
- In Fig. 1c, TβRII lane 3 and GAPDH lanes 2 and 3 appear highly similar to Fig. 3d hUASMC p-Smad3 lane 1 and β-actin (both lanes), respectively;
- In Fig. 4d, the images for the hUVEC and Scrambled hUVEC groups appear to contain overlap.

The authors have stated that these issues occurred due to errors in data storage. Additionally, they have been unable to provide the raw images of the western blots presented in Fig. 1c. The Editor-in-Chief therefore no longer has confidence in the presented data.

Jianzhong Xu has stated on behalf of all authors that none of the authors agree to this retraction.

